# The differential anti-tumour effects of zoledronic acid in breast cancer – evidence for a role of the activin signaling pathway

**DOI:** 10.1186/s12885-015-1066-7

**Published:** 2015-02-14

**Authors:** Caroline Wilson, Penelope Ottewell, Robert E Coleman, Ingunn Holen

**Affiliations:** 1 Academic Unit of Clinical Oncology, University of Sheffield, Medical School, Sheffield, UK; 2 Academic Department of Oncology, University of Sheffield, Medical School, Sheffield, UK

**Keywords:** Breast cancer, Zoledronic acid, Activin, Follistatin, Phosphorylated Smad2

## Abstract

**Background:**

Neo-adjuvant breast cancer clinical trials of zoledronic acid (ZOL) have shown that patients with oestrogen negative (ER-ve) tumours have improved disease outcomes. We investigated the molecular mechanism behind this differential anti-tumour effect according to ER status, hypothesising it may in part be mediated via the activin signaling pathway.

**Methods:**

The effects of activin A, its inhibitor follistatin and zoledronic acid on proliferation of breast cancer cells was evaluated using either an MTS proliferation assay or trypan blue. Secretion of activin A and follistatin in conditioned medium (CM) from MDA-MB-231, MDA-MB-436, MCF7 and T47D cell lines were measured using specific ELISAs. The effects of ZOL on phosphorylation domains of Smad2 (pSmad2c + pSmad2L) were evaluated using immunofluorescence. Changes seen *in vitro* were confirmed in a ZOL treated subcutaneous ER-ve MDA-MB-436 xenograft model.

**Results:**

Activin A inhibits proliferation of both ER-ve and oestrogen positive (ER + ve) breast cancer cells, an effect impaired by follistatin. ZOL significantly inhibits proliferation and the secretion of follistatin from ER-ve cells only, which increases the biological activity of the canonical activin A pathway by significantly increasing intracellular pSmad2c and decreasing nuclear accumulation of pSmad2L. *In vivo,* ZOL significantly decreases follistatin and pSmad2L expression in ER-ve subcutaneous xenografts compared to saline treated control animals.

**Conclusions:**

This is the first report showing a differential effect of ZOL, according to ER status, on the activin pathway and its inhibitors *in vitro* and *in vivo*. These data suggest a potential molecular mechanism contributing to the differential anti-tumour effects reported from clinical trials and requires further evaluation in clinical samples.

## Background

The addition of ZOL to neo-adjuvant chemotherapy has been shown to enhance the response of invasive breast cancer to chemotherapy [1]. However, not all breast tumours are equally responsive to the drug, with some studies suggesting that ZOL has a greater effect on primary tumour response and disease recurrence in patients with ER-ve, as opposed to ER + ve, tumours [2,3]. *In vitro*, ZOL inhibits proliferation and induces apoptosis of the ER-ve cell line MDA-MB-231, an effect not seen in the ER + ve cell line MCF7 [4]. The anti-tumour effects of ZOL reported from *in vitro* studies include reduced adhesion, migration and invasion of tumour cells, mediated by inhibition of farnesyl diphosphate (FPP) synthase and reduced prenylation of small GTPases (enzymes that hydrolyze guanosine triphosphate) [5].

The clinical neo-adjuvant breast cancer study, ANZAC, evaluated the biological effects of addition of ZOL to first cycle of FEC_100_ chemotherapy, and showed serum levels of follistatin significantly decreased following administration of ZOL in postmenopausal women [6]. Furthermore the addition of ZOL to chemotherapy reduced serum follistatin levels at day 5 post treatment specifically in patients with ER-ve tumours compared to patients receiving chemotherapy alone [7]. This may reflect a fall in the secretion of follistatin from ER-ve breast tumours that is not seen in ER + ve tumours.

**Figure 1:**
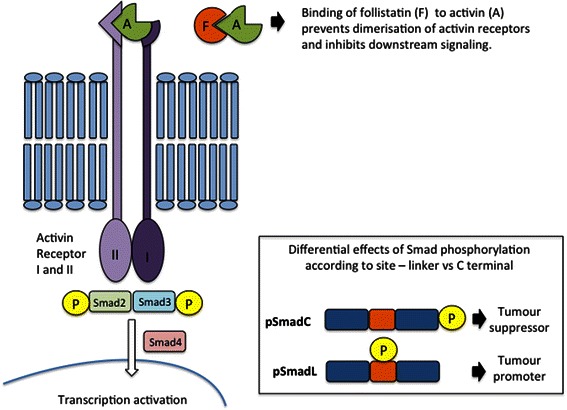
**The canonical activin pathway.** Activin binds to activin type II receptors resulting in phosphorylation of the C terminus of Smad2 (pSmad2C) or smad3 followed by nuclear translocation with co-receptor Smad4. Follistatin binds to activin preventing binding the type II receptor. Phosphorylation at the linker region of Smad2 or smad3 occurs downstream of cytoplasmic proteins such as RAS and nuclear proteins such as cyclin dependent kinases. The effector function of phosphorylated Smad2 is dependent on the site of phosphorylation; C terminus phosphorylation resulting in tumour growth suppression and linker phosphorylation resulting in tumour growth promotion.

Follistatin is a paracrine antagonist of activin and both proteins modify breast cancer cell proliferation. Activin is produced by breast cancer cells, inhibiting their proliferation, while follistatin binds to activin and prevents receptor binding with the type II receptor (ActRII), thus promoting proliferation [8]. Once activin binds to ActRII, dimerization occurs with ActRIB and the receptor associated intracellular proteins Smad2 and 3 are phosphorylated (Figure 1) [9]. Smad phosphorylation occurs either at the C terminus or at a linker region joining the MH1 and MH2 domains, with different effector functions; the C terminus being a tumour suppressor and the linker region being a tumour promoter [10] (Figure 1). ER-ve breast cancer cell lines have been shown to be insensitive to the anti-proliferative effects of activin [11], however this effect does not appear to be due to low expression of the activin type II receptor, with evidence that MDA-MB-231 express activin type II receptors [11] and MDA-MB-436 have a functional activin-signaling pathway showing phosphorylation of Smad2 in response to exogenous activin following removal of follistatin from the medium [12]. These data indicate that exogenous neutralisers of activin, i.e. follistatin, are responsible for the lack of inhibition of proliferation in response to activin in ER-ve cell lines, rather than absence of/non functional activin receptors.

We provide the first evidence that ZOL can affect the activin signaling pathway specifically in ER-ve breast cancer cell lines by a dual mechanism; decreasing secretion of follistatin and preventing nuclear localization of linker phosphorylated Smad2.

## Methods

### Cell lines and reagents

ER-ve (MDA-MB-231, MDA-MB-436) and ER + ve (MCF7, T47D) human breast cancer cells were purchased from European Collection of Cell Lines and routinely cultured in RPMI + 10% foetal calf serum (FCS). Evaluation of secretion of proteins from cell lines into conditioned medium (CM) and effects on pSmad2C was performed using human activin A and follistatin quantikine ELISAs and the cell based phospho-Smad2/3 fluorescent ELISA, purchased from R&D systems (Oxford, UK). Cell titre 96 Aqueous One solution cell proliferation assay (MTS) was purchased from Promega (Southampton, UK). The tumour samples were obtained from MDA-MB-436 previously described xenograft studies [13]. Recombinant human activin A and follistatin were purchased from R&D systems (Oxford, UK). ZOL ([(1-hydroxy-2-(1H-imidazol-1-yl) ethylidene] bisphosphonic acid) was supplied as the hydrated di-sodium salt by Norvartis Pharma (Basel, Switzerland). Primary antibodies were purchased from Santa Cruz USA (Rap1a), Abcam UK (GAPDH) and Cell Signaling UK (phosphoSmad2, all secondary antibodies). SB-431-542 was purchased from Tocris bioscience (Bristol, UK).

### Western blotting

Cells were lysed in cell lysis buffer (Sigma-Aldrich) and proteins were resolved using 12% SDS-PAGE. Proteins were immobilized on polyvinylidene difluoride (PVDF) membrane, blocked (5% milk) and probed with antibodies specific to unprenylated Rap1a (1:200), pSmad2L (1:1000), with GAPDH (1:20,000). Representative blots from three separate experiments are shown.

### Enzyme linked immunoabsorbance assays

Human Follistatin and Activin A ELISAs were carried out according to the manufacturers instructions using CM from tumour cells. Minimum detection limits were 29 pg/ml and 3.67 pg/ml, respectively, with intra-assay CVs <15%. Molar ratios were calculated as follows; mean CM concentrations (pmol/L) divided by molecular weight of the protein, expressed as a ratio (follistatin:activin).

The quantification of total smad2/3 to phosphoSmad2/3 was carried out using a cell based phospho-Smad2/3 fluorescent ELISA. 1.5x10^3^ MDA-MB-231 cells were seeded in a 96 well plate and the ELISA was carried out according to the manufacturers instructions.

### Proliferation assay

Cell proliferation was assessed either using an MTS assay or viable cell counting with trypan blue. For the MTS assay 1.5x10^3^ MDA-MB-231/3x10^3^ MCF7 cells were seeded in 96 well plates and for the trypan blue assay 1x10^5^ cells were seeded in 6 well plates. Cells were serum starved for 24 hours before addition of recombinant protein/drug in RPMI + 10%FCS, cells were washed and protein/drug replaced every 24 hours. At completion of the MTS assay 20 μl of MTS solution was added directly to each well and quantified on a plate reader. At completion of the trypan blue assay cells were trypsinised to remove from wells and trypan added in a 50:50 concentration of cell suspension to trypan blue and counted using a haemocytometer.

### Immunofluorescence

To visualize pSmad2C and pSmad2L, 2x10^4^ MDA-MB-231/4x10^4^ MCF7 cells were seeded in chamber slides, serum starved for 24 hours and then treated for 48 hours with ZOL (50 μM). Cells were fixed (4% paraformaldehyde) and blocked (5% goat serum) prior to incubation with phosphoSmad2 antibodies (1:100). After incubation with secondary antibodies (1:100) and fluorescein-avidin, coverslips were mounted with DAPI and viewed on an inverted fluorescent light microscope. Images of ≥100 cells per chamber manually scored for nuclear localization using the blue dapi counter stain as a nuclear localiser.

### Immunohistochemical staining for follistatin and pSmad2L

Paraffin embedded tumour sections from previously published *in vivo* experiments were used [13]. The *in vivo* experimental design used female MF1 nude mice injected with 5x10^5^ MDA-MB-436 cells sub-cutaneous and animals were treated weekly with 100 μg/kg intra peritoneal ZOL vs. saline control for 6 weeks starting on day 7. Tumours were processed for histology using standard protocols. Sections were dewaxed, blocked (3% H_2_O_2_ in methanol) for 10 minutes followed by trypsin antigen retrieval for 15 minutes at 37°C. Further blocking (5% goat serum/1% bovine serum albumin) for 30 minutes was followed by addition of primary antibody overnight (pSmad2L 1:100/follistatin 1:200). Secondary antibodies were added (1:200) for 30 minutes followed by a 6-minute incubation with DAB. All animal experiments were carried out in accordance with local guidelines and with Home Office approval under project license 40/2343 held by Professor N. J. Brown, University of Sheffield, UK.

### Statistical analysis

Unless stated otherwise, all experiments were carried out with 3 replicates and 3 repeats. Prism GraphPad (5.0a) was used for statistical analysis. Data analysis was by non-parametric Mann–Whitney test to compare differences between groups or Wilcoxon Signed-Rank test to compare related groups. Data represent mean and SEM. Statistical significance is defined as a p value = <0.05.

## Results

### Activin A inhibits the proliferation of ER + ve and ER-ve breast cancer cells

**Figure 2:**
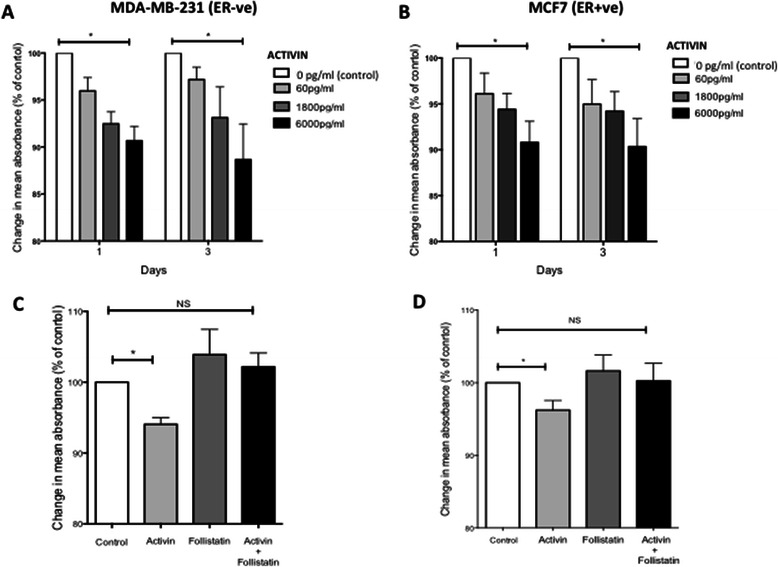
**Activin inhibits breast cancer cell proliferation.** MDA-MB −231 **(A + C)** and MCF7 **(B + D)** cells were treated with increasing doses of activin in a timecourse experiment **(A + B)**, or with recombinant activin (6000 pg/ml) +/− follistatin (64,000 pg/ml) for 72 hours **(C + D)**. 20 μl of MTS solution was added 4 hours prior to the final time points to evaluate absorbance of treated cells relative to control untreated cells. Data represents mean + SEM of 8 replicates and 5 repeats. Wilcoxon Signed-Rank test for significance, *p value <0.05, NS not significant.

Previous reports have shown that proliferation of ER + ve cells is inhibited by activin [14], but the effect of activin on proliferation of ER-ve cell lines is less clear [11]. In order to compare the effect of activin and follistatin on proliferation in ER-ve and ER + ve cell lines, a time course and dose response MTS assay was performed. Both the ER-ve (MDA-MB-231) and ER + ve (MCF7) cells showed a significant decrease in proliferation compared to control following addition of activin A on days 1 and 3 (Figure 2A + B) which was lost by day 5 (data not shown). There was a significant dose-dependent inhibition of proliferation with increasing doses of activin A in both cell lines. These results show that both ER-ve and ER + ve cell lines are responsive to the growth inhibitory effect of activin A. To confirm the decrease in proliferation was occurring in an activin dependent manner in ER-ve cell lines given the controversy in the literature, MDA-MB-231 cells were treated with activin 6000 pg/ml +/− an ALK4/5 inhibitor SB-431-542 (10 μM/l) for 72 hours. The ALK4/5 inhibitor will prevent ActRII dimerising with its type I receptor ALK4. MDA-MB-231 cells showed less inhibition of proliferation with addition of the ALK4/5 inhibitor to activin (mean % change in absorbance from control Activin; −11%, Activin + ALK4/5 inhibitor −3.4%) confirming the changes in proliferation in ER-ve cells was activin-dependent (data not shown).

### Follistatin impairs the inhibition of proliferation induced by activin A

Follistatin is reported to negate the anti-proliferative effect of activin [12]. In order to evaluate the effect of follistatin in ER-ve (MDA-MB-231) and ER + ve (MCF7) cells they were treated with 6000 pg/ml of activin A in the presence or absence of follistatin (64,000 pg/ml) for 72 hours (activin concentrations were chosen to replicate inter-tumoural levels of activin in breast tumours [15]). The significant inhibitory effect of activin A on cell proliferation was negated in the presence of follistatin in MDA-MB-231 cells (mean % change from control; Activin −6% [SEM 0.92], Activin + follistatin +2.6% [SEM 1.9]). In MCF7 cells a similar, but non-significant, trend was also seen (mean % change from control; Activin −3.2% [SEM 1.3], Activin + follistatin −0.02% [SEM 2.4]) (Figure 2C + D). These data provided further indication that ER-ve cell lines are sensitive to the growth inhibitory effects of activin A and that this effect is inhibited by follistatin.

### ER-ve cells secrete more activin A and follistatin than ER + ve cells

**Figure 3:**
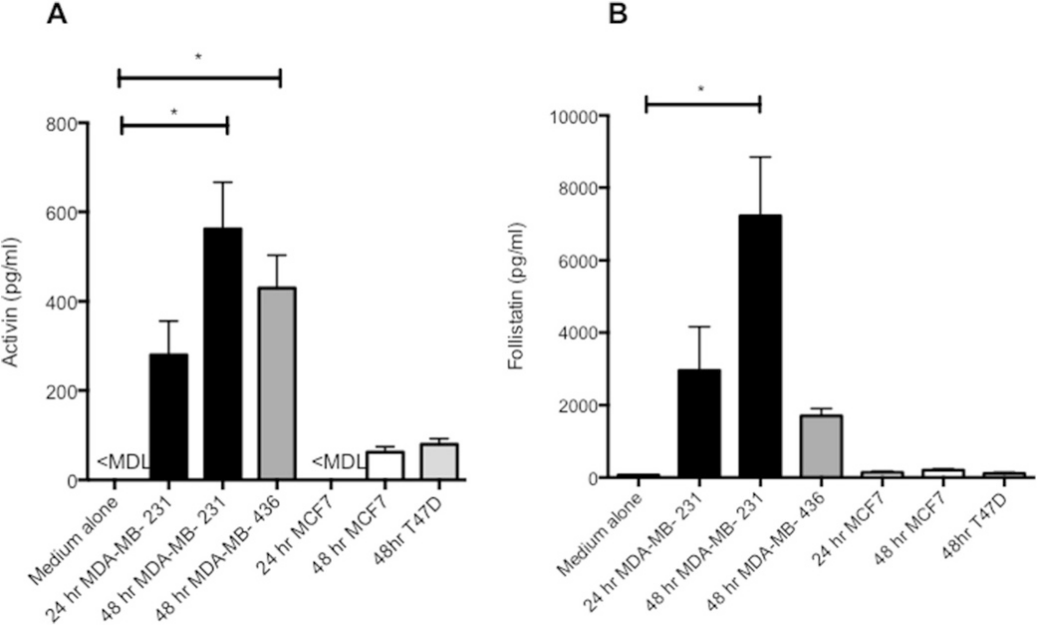
**Activin and follistatin secretion from ER- breast cancer cell lines and ER + ve breast cancer cell lines.** 1x10^5^ MDA-MB-231 or MDA-MB-436 (ER-ve) and 4x10^5^ MCF7 or T47D (ER + ve) cell lines were plated in 6 well plates and levels of Activin **(A)** and follistatin **(B)** in the medium determined by ELISA at 24 and 48 hours. Data represents 3 replicates and 3 repeats. Mann Whitney test for significance comparing wells with cells to media alone (no cells), *p value <0.05. <MDL = below assay minimum detection limit.

The quantity of activin A and follistatin secreted from ER-ve (MDA-MB-231 and MDA-MB-436), and ER + ve (MCF7 and T47D) cells was determined by ELISA. As shown in Figure 3A, both ER-ve cell lines secreted significant levels of activin A (MDA-MB-231 = 561 pg/ml [SEM 104.9] p value = 0.0034, MDA-MB-436 = 430 pg/ml [SEM 73.8] p value = 0.0436). Follistatin was detectable in the medium from all cell lines, although the levels were much lower in ER + ve cell lines (mean level pg/ml; MCF7 = 200, T47D = 108) compared to ER-ve cell lines (mean level pg/ml; MDA-MB-231 = 7224, MDA-MB-436 = 1704 Figure 3B). These results suggest that ER-ve cells could be more dependent on activin A for regulation of cell growth than ER + ve cells, and may utilise secretion of follistatin as a mechanism of escape from the anti-proliferative effects of activin A. There is no clear evidence to indicate why tumor cells secrete a growth inhibitor such as activin [16], suggesting it may be have alternative functions that the cells escape from by alternative mechanisms i.e. secretion of inhibitors or down regulation of receptors.

### Zoledronic acid differentially affects proliferation of breast cancer cell lines according to ER status

**Figure 4:**
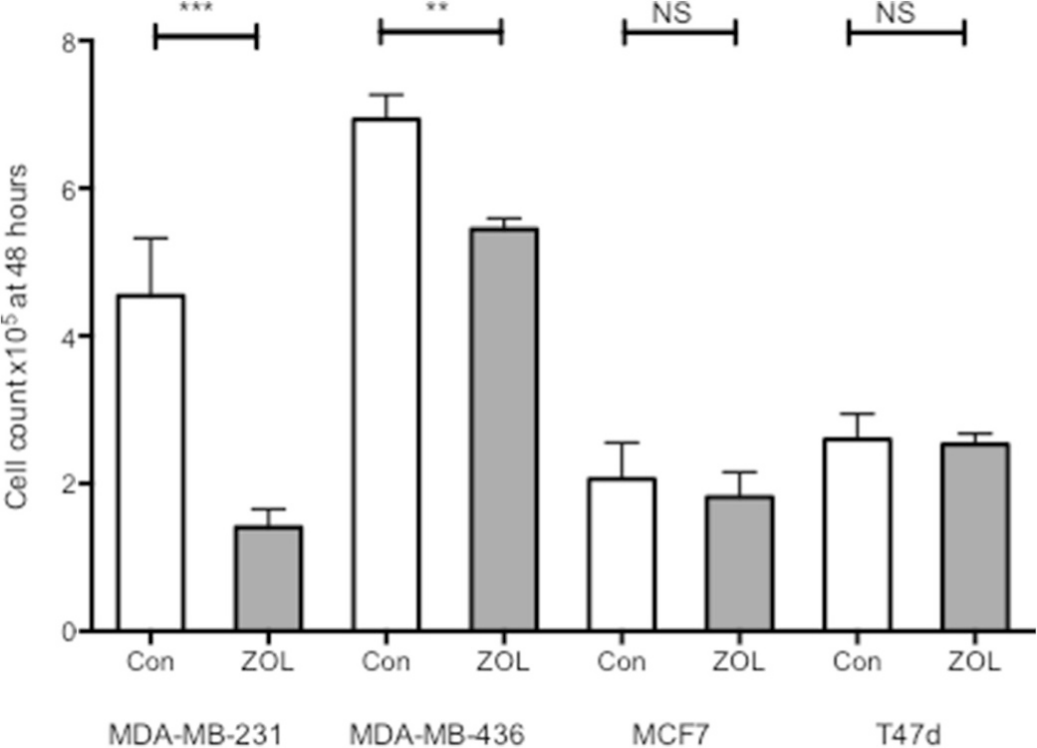
**Effects of zoledronic acid on proliferation of ER + ve and ER-ve cell lines.** 1x10^5^ MDA-MB-231, MDA-MB-436 (ER-ve),MCF7 or T47D (ER + ve) cell lines were plated in 6 well plates. Cell were treated for 48 hours with medium +/− 50 μM ZOL. At 48 hours viable cell count was performed using trypan blue. Data represents 3 replicates and 3 repeats. Mann Whitney test for significance comparing control with ZOL treated, NS not significant, **p value < 0.005, ***p value <0.0005.

To evaluate if zoledronic acid could affect proliferation of ER + ve and ER-ve breast cancer cell lines MDA-MB-231, MDA-MB-436, T47D and MCF7 cells were treated with 50 μM ZOL or medium control for 48 hours and live cell count performed with trypan blue. ZOL significantly inhibited proliferation of both ER-ve cell lines compared to control (Cell count x10^5;^ MDA-MB-231 control 3.4 [SEM 0.79], ZOL 1.6 [SEM 0.25] p value 0.0009, MDA-MB-436 control 6.9 [SEM 0.33] ZOL 5.3 [SEM 0.14] p value 0.0043), but did not significantly alter proliferation of the ER + ve cell lines MCF7 and T47D (Figure 4).

### Zoledronic acid decreases follistatin secretion from ER-ve cell lines only

**Figure 5:**
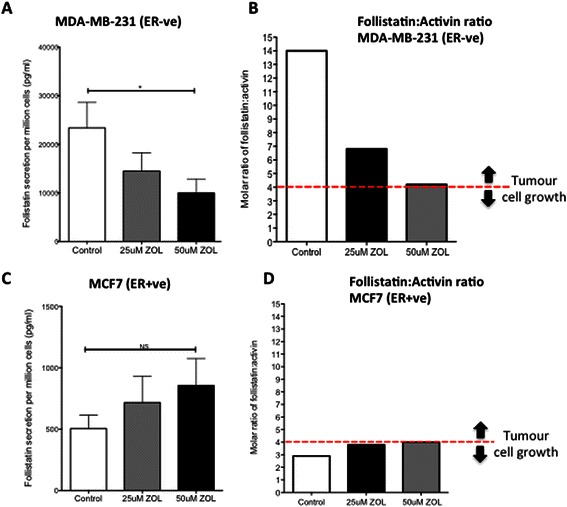
**Effects of zoledronic acid on follistatin secretion and follistatin:activin ratio.** MDA-MB-231 **(A)** and MCF7 **(C)** cells were treated with medium alone, 25 μM or 50 μM ZOL for 48 hours and levels of secreted activin and follistatin measured by ELISA. Molar ratio of follistatin:activin **(B + D)** was calculated by converting mean quantity of secreted protein per million cells (pg/ml) to pmol/l by dividing by the molecular weight of each protein, and then expressed as a ratio. A molar ratio of 4 (dashed line) represents the level at which activin is neutralised by follistatin: an excess of follistatin:activin increases tumour growth (above dashed line). Data represents mean + SEM of 3 replicates and 3 repeats, *= p value <0.05, NS not significant.

To investigate if ZOL could affect the secretion of activin A and/or follistatin from MDA-MB-231 and MCF7 cells, both cell lines were exposed to medium +/− 25 μM/50 μM ZOL for 48 hours. Activin A secretion was unaffected by ZOL in either cell line. In contrast, follistatin secretion was significantly decreased in MDA-MB-231 cells after exposure to ZOL (control 23378 pg/ml [SEM 5259], ZOL 9987 pg/ml [SEM 2871], p value =0.0012), and also fell in MDA-MB-436 cells (control 1928 pg/ml [SEM 188], ZOL 1592 pg/ml [SEM 65] p value 0.07), but did not change in MCF7 (Figure 5) or T47D cells (data not shown). We hypothesise that the biological activity of activin A depends on the ratio of activin A to follistatin in the tumour microenvironment. The literature reports a 4:1 molar ratio of follistatin:activin would neutralize activin [17]. ZOL reduced the molar ratio of follistatin:activin secreted by MDA-MB-231 cells (control ratio 14:1, ZOL ratio 4:1) (Figure 5B), compared to minimal change in MCF7 cells (control ratio 3:1, ZOL ratio 4:1) (Figure 5D) indicating ZOL has a more noticeable effect on the follistatin:activin ratio in ER-ve cell lines.

**Figure 6:**
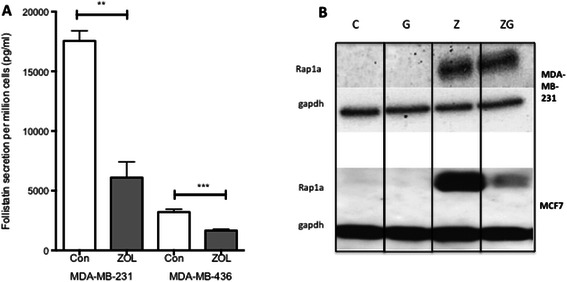
**Effects of pulsed zoledronic acid on follistatin secretion from ER-ve cell lines. The difference in follistatin secretion according to ER status is not due to differences in cellular uptake of the drug. A**. MDA-MB-231 and MDA-MB-436 cells were treated for 4 hours with 50 μM of ZOL followed by a 44 hour incubation with medium alone or medium alone for 48 hours (CON). Follistatin levels in the supernatant was removed and processed for ELISA. Data represents mean + SEM of 3 replicates and 3 repeats, **= p value <0.01, ***= p value <0.001. **B**. MDA-MB-231 and MCF7 cells were treated for 48 hours with medium alone (C), GGOH 50 μM (G), Zol 10 μM (Z) or both G and Z in combination (ZG). Rap1a antibody (1:200) used to assess levels of unprelylated protein and GAPDH (1:20,000) used as loading control.

To evaluate the effects of a short (clinically achievable) exposure of ZOL on follistatin secretion in ER-ve cell lines, MDA-MB-231 and MDA-MB-436 cells were treated with 50 μM ZOL for 4 hours, followed by 44 hours incubation in drug-free medium. The secretion of follistatin was significantly decreased in both cell lines by a 4-hour pulse of ZOL (mean follistatin pg/ml, MDA-MB-231; control = 17551 [SEM 847], ZOL = 6106 [SEM 1315] p value =0.0015. MDA-MB-436; control = 3209 [SEM 236], ZOL =1667 [SEM 116] p value = 0.001) (Figure 6A). These data show that even a short exposure to ZOL decreases follistatin secretion from ER-ve cell lines.

### Both ER + ve and ER-ve cell lines take up ZOL *in vitro*

ZOL increases accumulation of unprenylated small GTPases i.e. Rap1a via inhibition of the mevalonate pathway [18]. The lack of effect of ZOL on follistatin secretion in the ER + ve cells was considered to possibly reflect a limited drug uptake. To evaluate if the ER + ve and ER-ve cell lines used in this study had a similar levels of ZOL uptake we used western blotting to compare the accumulation of unprenylated Rap1a (uRap1a, a surrogate marker of ZOL uptake) in MCF7 and MDA-MB-231 cells treated with ZOL, and if addition of the mevalonate pathway intermediary, geranylgeraniol (GGOH), could inhibit the accumulation of uRap1a. Both cell lines had increased levels of uRap1a in response to treatment with ZOL that was partially reversed by addition of GGOH (Figure 6B). These data suggest that the difference in follistatin secretion between ER-ve and ER + ve cell lines is not due differential cellular uptake of ZOL.

### Zoledronic acid reduces intracellular C terminus phosphorylated Smad2

**Figure 7:**
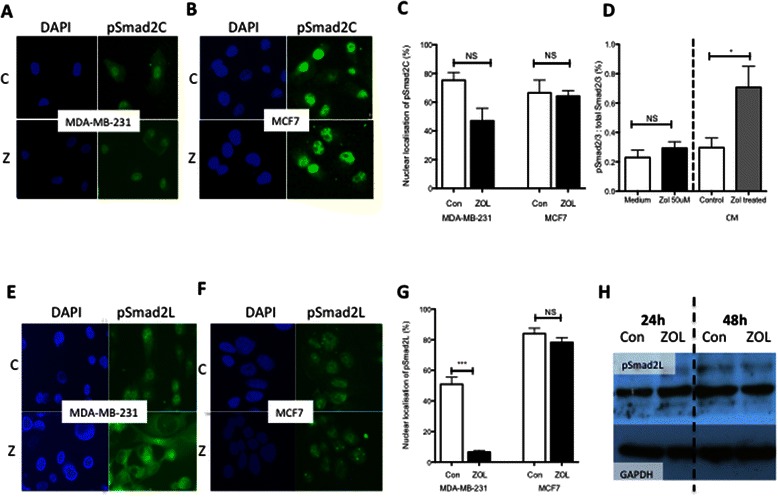
**Smad2 phosphorylation at both c terminus and linker region is differentially affected by zoledronic acid according to ER status of breast cancer cells. A + B** Representative immunofluorescent images of pSmad2C (green) and dapi staining (blue) in MDA-MB-231 cells **(A)** and MCF7 cells **(B)** treated with medium alone (C) or ZOL (Z) for 48 hours. **C**. Quantification of nuclear localisation of pSmad2C in MDA-MB-231 and MCF7 cells treated for 48 hours with medium alone (con) or ZOL (50 μM). Data represents minimum 100 cells per group, Mann Whitney U test for significance, NS = not significant **D**. Ratio of total cellular quantity of total Smad2/3 to pSmad2/3 in MDA-MB-231 cells treated for 1 hour with medium alone, ZOL (50 μM) or conditioned medium (CM) from MDA-MB-231 cells previously treated with ZOL (50 μM) or medium (control). Data represents 3 replicates and 3 repeats, Mann Whitney U test for significance, NS = not significant, *p = <0.05 **E + F** Representative immunofluorescent images of pSmad2L (green) and dapi staining (blue) in MDA-MB-231 cells **(E)** and MCF7 cells **(F)** treated with medium alone (C) or ZOL (Z) for 48 hours. **G**. Quantification of nuclear localisation of pSmad2L in MDA-MB-231 and MCF7 cells treated for 48 hours with medium alone (con) or ZOL (50 μM). Data represents minimum 100 cells per group, Mann Whitney U test for significance, NS = not significant, ***p value <0.001. **H**. Representative western blots for cellular quantity of pSmad2L and gapdh in MDA-MB-231 cells treated with medium alone (con) or ZOL (50 μm).

To evaluate if the activin-signaling pathway downstream of surface receptors is affected by ZOL, localization and intracellular quantity of pSmad2C in MDA-MB-231 and MCF7 cells was assessed. Using an immunofluorescence method, we detected no significant difference in the percentage of cells with nuclear localization of pSmad2C after treatment with ZOL compared to control in either cell line (Figure 7A-C). However, MDA-MB-231 cells exposed to CM from ZOL treated cells (containing low levels of follistatin) showed significantly higher levels of pSmad2C than cells exposed to CM from medium only treated cells (control = 0.29 [SEM 0.065], ZOL = 0.7 [SEM 0.14], p value 0.0286) (Figure 7D). This effect was not seen in MDA-MB-231 cells exposed directly to 50 μM ZOL, indicating that the decreased follistatin levels in CM from ZOL treated cells was responsible for the increase in intracellular levels of pSmad2C.

### Zoledronic acid decreases nuclear localization of linker phosphorylated Smad2

Whereas pSmad2C is recognized to function as a tumour suppressor in breast cancer [19], pSmad2L may act as a tumour growth promoter [10]. We evaluated if ZOL could affect cellular localization of pSmad2L in MDA-MB-231 and MCF7 cells using immunofluorescence. The percentage of MDA-MB-231 cells with nuclear localization of pSmad2L was significantly decreased after treatment with ZOL (control = 50% [SEM 4.6], ZOL =6.6% [SEM 1.1], p value <0.0001). No significant difference was seen between ZOL and control in the MCF7 cells (Figure 7E-G). Using western blotting we found that ZOL did not cause a significant alteration in the total cellular levels of pSmad2L, suggesting that ZOL alters cellular localization of pSmad2L in MDA-MB-231, but not the total quantity (Figure 7H).

### Zoledronic acid decreases follistatin and pSmad2L expression in an ER-ve xenograft model

**Figure 8:**
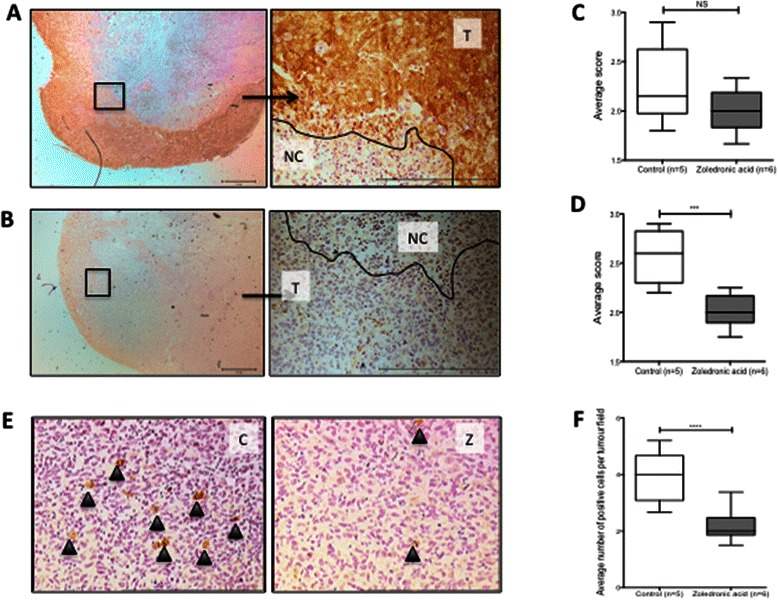
**Follistatin and pSmad2L expression in MDA-MB-436 xenografts is reduced following zoledronic acid treatment*in vivo*. A**. Representative images of follistatin expression in tumours from saline treated mice at x1.6 magnification (left) and x20 magnification (right). Viable tumour cells (T), necrotic core of tumours (NC). **B** Representative images of follistatin expression in tumours from ZOL treated mice. **C + D**. 20 x 750 μm^2^ images were scored from two sections per tumour. Images were scored for intensity of + ve stain **(C)** and area of + ve stain **(D)**. Data represents the mean scores + SEM. Mann Whitney U test for significance, ***p value <0.001, NS not significant. **E** Representative images of pSmad2L expression (black arrows) in saline treated mice (C) and ZOL treated mice (Z). **F** 20x750μm^2^ images were scored from two sections per tumour. Number of positive cells were counted and data represents mean scores + SEM. Mann Whitney U test for significance, ***p value <0.001.

In order to validate that ZOL induces changes in follistatin and pSmad2L in ER-ve tumours *in vivo*, ER-ve MDA-MB-436 sub-cutaneous tumour sections from mice treated with or without ZOL (100 μg/kg, weekly for 6 weeks, equivalent to the 4 mg clinical dose) were evaluated. Follistatin expression was scored for intensity and area of positive stain. Data were analysed using average scores from 2 assessors blinded to the treatment groups. No difference was seen in the intensity of follistatin staining, however, there was a significant decrease in the tumour area staining positive for follistatin in mice treated with ZOL compared to saline (Figure 8A-D). The number of cells with mitotic nuclei staining positive for pSmad2L was significantly lower in tumours from ZOL treated mice compared to saline treated (Figure 8E-F). These results suggest that using the dosing regime described, ZOL can directly alter expression of both follistatin and pSmad2L in ER-ve subcutaneous tumours *in vivo*.

## Discussion

In this study we describe a novel anti-proliferative mechanism of action of ZOL in ER-ve breast cancer cells, involving the activin-signaling pathway, and suggest that this may contribute to the enhanced anti-tumour effect of ZOL in ER-ve breast cancers demonstrated in neo-adjuvant clinical trials [2,20].

In agreement with published data, we found that activin A inhibits proliferation of ER + ve MCF7 cells [21]. However, we saw a very similar inhibition of growth in ER-ve MDA-MB-231 cells, in contrast to previously reported data [10]. Kalkoven et al. suggested the mechanism responsible for resistance to the anti-proliferative effects of activin A was located downstream of the receptor [11], however, alternative mechanisms such as the presence and/or effect of secreted activin neutralizers like follistatin was not evaluated.

Breast cancer cells have been shown to express the follistatin related gene (FLRG), encoding follistatin and follistatin related protein [12]. This same study also demonstrated that the anti-proliferative effect of activin was weak in MCF7 and undetectable in MDA-MB-436 cells. However, when endogenous secreted inhibitors i.e. follistatin were removed, Smad2C was phosphorylated in response to activin in both cell lines, and silencing of FLRG increased levels of pSmad2C and decreased proliferation in response to endogenous activin. These results support our data, demonstrating that activin inhibitors such as follistatin can neutralise the anti-proliferative effects of activin in both ER-ve and ER + ve cell lines.

We found that both activin A and follistatin were secreted from ER-ve and ER + ve cells, but at different levels, hence generating different effects on tumour cell proliferation. ER-ve cells secreted an excess of follistatin:activin, favouring cell proliferation, and in contrast to ER + ve cells which secreted an excess of activin:follistatin, favouring growth suppression. Previously published data have demonstrated that the activin βA subunit is detected in higher levels in breast carcinoma compared to normal breast tissue [15], and activin type II cell surface receptors were attenuated with increasing tumour grade [22]. These studies did not include measurements of follistatin expression. It is possible that resistance to the tumour suppressive actions of activin in breast cancer is linked to the levels of secretion of activin neutralizing molecules such as follistatin, as well as a concurrent decrease in expression of activin type II receptors. This potential mechanism requires exploration in clinical neo-adjuvant breast cancer studies.

The differential effect of ZOL on follistatin secretion according to ER status of breast cancer cell lines *in vitro* and *in vivo* demonstrated in this study, has not been previously reported. However, other studies have shown a differential effect of ZOL on proliferation according to ER status. Rachner *et al.* demonstrated that MCF7 cells did not alter proliferation rates in response to ZOL, whereas MDA-MB-231 cells showed a significant dose-dependent inhibition of proliferation and increase in apoptosis via activation of caspase 3 and 7 [4]. We detected uRap1a in both MCF7 and MDA-MB-231 cells after treatment with ZOL, suggesting that variable uptake of the drug is not an explanation for the differing effects on follistatin. This is in agreement with a report by Monkkonen *et al.*, showing accumulation of uRap1A and isopentenyl diphosphate (IPP) in both MCF7 and MDA-MB-436 cells following 24 h treatment with 25 μM ZOL [23]. However, uptake of ^14^C-labelled ZOL is reported to be 3 fold lower in ER-ve BO2 cells compared to ER + ve T47D and MCF-7 cells one hour after addition of 25 μM ZOL [24]. These contrasting results may be due to the differences in dose and time of exposure to the drug. Whether the ZOL-induced reduction in follistatin secretion from ER-ve cells is due to a direct effect of the drug on the mevalonate pathway remains to be established.

We found that the decrease in follistatin secretion from MDA-MB-231 cells affected the downstream protein Smad2, increasing the levels of pSmad2/3C relative to total Smad2/3. Phosphorylation of Smad2 at the C terminus domain has been shown to suppress breast cancer cell invasion and metastases to bone *in vivo*. In a mouse model of bone metastasis, Smad2 knockdown in MDA-MB-231 cells resulted in significantly faster tumour establishment in bone compared to the parental cell line, suggesting a tumour suppressive role [25]. In clinical studies, a tissue microarray study of breast tumours from 426 patients showed that loss of pSmad2C was associated with a shorter median overall survival (110.5 vs. 306.5 weeks, p = 0.024), suggesting that this may be a tumour specific poor prognostic factor [19]. Moreover, phosphorylation of Smad2 at the linker region has been reported to alter its action from tumour suppression to tumour promotion. Phosphorylation at this site is primarily via cytoplasmic RAS and nuclear cyclin dependent kinases [10], as opposed to the canonical receptor-mediated activin pathway leading to C terminus phosphorylation. Small GTPases have been found to affect the activin signaling pathway, with Rap2 increasing activin cell surface receptor expression, potentially increasing cellular responses to endogenous and exogenous activin [26]. However, RAC1 has been shown to inhibit smad2/3 activation [27], suggesting small GTPases can have differential effects on the activin pathway. We showed that ZOL decreased nuclear localization of pSmad2L in ER-ve breast cancer cells *in vitro* and the number of cells staining positive *in vivo*. Whether this effect of ZOL is via a small GTPase/RAS dependent mechanism remains to be confirmed, but ZOL has been shown to decrease RAS expression and activity in ER-ve cell lines (MDA-MB-231 and BRC-230), with inhibition of cell proliferation [28].

## Conclusion

Taken together, our data support a potential dual mechanism of action of ZOL on the activin signaling pathway in ER-ve breast cancer cells *in vitro* and *in vivo*; firstly via a decrease in follistatin secretion leading to an increase in the tumour suppressor pSmad2C, and secondly via a decrease in nuclear localization of the tumour promotor pSmad2L*.* These data provide a possible novel direct anti-proliferative mechanism of action of ZOL on breast cancer cells involving activin signaling, that could contribute to the enhanced anti-tumour effects of the drug in neo-adjuvant clinical trials of patients with ER–ve breast cancer, and requires further research in clinical samples. The potential indirect anti-proliferative mechanism of action of ZOL on breast cancer cells in the bone microenvironment involving activin signaling also requires further investigation, and may contribute preclinical data to explain the results of clinical adjuvant bisphosphonate trials where the burden of residual disease is likely to be within niches such as the bone.
